# INTENTION TO USE WEARABLE ROBOTS FOR INDEPENDENT LIVING : THE ROLE OF CULTURE AND PERSUASIVE HEALTH COMMUNICATION

**DOI:** 10.1093/geroni/igad104.1300

**Published:** 2023-12-21

**Authors:** Clio Yuen Man Cheng, Vivian Weiqun Lou

**Affiliations:** The University of Hong Kong, Hong Kong, Hong Kong; The University of Hong Kong, Hong Kong, Hong Kong

## Abstract

Soft wearable robots are emerging assistive technologies that can prolong independent living among older adults experiencing progressive muscle loss, such as sarcopenia. In order to fulfil the wish of aging-in-place and relieve caregiver burden, it is essential to increase wearable robots acceptance through persuasive health communication strategies. This study draws on the integration of the senior technology acceptance model, health communication, and cultural value orientation to predict the intention to use wearable robots among older adults. A 2 (message framing: gain- versus loss-framed) x 2 (temporal framing: distal- versus proximal-framed) randomized experiment was conducted with 154 older adults aged 50 to 74 years, using a self-administered cross-sectional from December 2022 to March 2023 in Hong Kong. A hierarchical multiple regression analysis was conducted with six blocks of variables. Demographic variables including age, gender, and sarcopenia potential were not significantly associated with intention to use. Horizontal individualism is significantly associated with intention to use (p=.011). Also, horizontal collectivism is significantly associated with intention to use (p=.018). Vertical collectivism interacts with persuasive health communication to predict intention to use among older adults (p=.041). The final model (F(12,141) = 3.699, p<.001, R2=.24) which included cultural value orientations showed significant improvement from the first model which only included age and gender. Overall, the final model accounted for 23.9% of the variance. This study is the first to predict the intention to use wearable robots among older adults and provide valuable insights on future policy development to promote aging-in-place, with the aid of wearable robots.

